# Arabidopsis *LEC1* and *LEC2* Orthologous Genes Are Key Regulators of Somatic Embryogenesis in Cassava

**DOI:** 10.3389/fpls.2019.00673

**Published:** 2019-05-22

**Authors:** Alejandro Brand, Mauricio Quimbaya, Joe Tohme, Paul Chavarriaga-Aguirre

**Affiliations:** ^1^International Center for Tropical Agriculture, Cali, Colombia; ^2^Department of Natural Sciences and Mathematics, Pontificia Universidad Javeriana Cali, Cali, Colombia

**Keywords:** cassava, somatic embryogenesis, leafy cotyledon (LEC), LEC1, LEC2, transcription factors, embryogenesis promoting factors (EPFs)

## Abstract

High genotype-dependent variation in friable embryogenic callus (FEC) induction and subsequent somaclonal variation constitute bottlenecks for the application and scaling of genetic transformation (GT) technology to more farmer- and industry-preferred cassava varieties. The understanding and identification of molecular factors underlying embryogenic development in cassava may help to overcome these constraints. Here, we described the *Arabidopsis thaliana* LEAFY COTYLEDON (LEC) *LEC1* and *LEC2* orthologous genes in cassava, designated as *MeLEC1* and *MeLEC2*, respectively. Expression analyses showed that both, *MeLEC1* and *MeLEC2*, are expressed at higher levels in somatic embryogenic (SE) tissues in contrast with differentiated mature tissues. The rapid expression increase of *MeLEC* genes at early SE induction times strongly suggests that they are involved in the transition from a somatic to an embryonic state, and probably, in the competence acquisition for SE development in cassava. The independent overexpression of the *MeLEC* genes resulted in different regenerated events with embryogenic characteristics such as *MeLEC1^OE^* plants with cotyledon-like leaves and *MeLEC2^OE^* plants with somatic-like embryos that emerged over the surface of mature leaves. Transcript increases of other embryo-specific regulating factors were also detected in *MeLEC^OE^* plants, supporting their mutual interaction in the embryo development coordination. The single overexpression of *MeLEC2* was enough to reprogram the vegetative cells and induce direct somatic embryogenesis, which converts this gene into a tool that could improve the recovery of transformed plants of recalcitrant genotypes. The identification of *MeLEC* genes contributes not only to improve our understanding of SE process in cassava, but also provides viable alternatives to optimize GT and advance in gene editing in this crop, through the development of genotype-independent protocols.

## Introduction

Cassava (*Manihot esculenta* Crantz) is a root crop that provides staple food for an estimated 800 million people worldwide (Howeler et al., 2013). In many countries of sub-Saharan Africa, cassava is the cheapest source of calories especially for small-holder, low-income farmers who grow it with limited external inputs and under suboptimal conditions (Howeler et al., 2013). Likewise, cassava has a growing impact on the industry that uses the roots as a raw material for processed food and biofuels (Ceballos et al., 2012). Given the high economic and social impact of cassava production, its genetic improvement is a must, nevertheless, its high heterozygosity, long life cycle, unsynchronized flowering and inbreeding depression, constrain conventional breeding (Ceballos et al., 2004). The implementation of genetic transformation (GT) and gene editing (GE) technologies in cassava has brought important contributions for its improvement, accelerating the incorporation of new traits like increase in iron and zinc (Narayanan et al., 2019) and the reduction of cassava brown streak disease (CBSD) symptoms (Gomez et al., 2019), respectively. However, after more than two decades of cassava GT advances, difficulties in tissue culture and plant regeneration methods are still limiting the transferring of these technologies from few models, proof-of-concept genotypes to farmer- and industry-preferred varieties (Chavarriaga-Aguirre et al., 2016).

*Agrobacterium*-mediated GT in combination with friable embryogenic callus (FEC) is, up to date, the most efficient and widely used method for DNA delivery (Taylor et al., 1996; Bull et al., 2009; Liu et al., 2011). Although this protocol is fairly standardized for the benchmark cv. 60444 (Taylor et al., 2012), its response capacity varies significantly between genotypes (Liu et al., 2011; Rey and Vanderschuren, 2017). Several steps and sub-culturing procedures make FEC production a highly resource demanding and time-consuming task, in addition to the low-quality regeneration frequencies and high somaclonal variation (Raemakers et al., 2001; Taylor et al., 2001; Bull et al., 2009, 2011; Zainuddin et al., 2012; Ma et al., 2015). Recently efforts have been conducted to enhance FEC induction and transformation of more cassava cultivars (reviewed by Zhang et al., 2017). Nevertheless, in most cases, the efficiency needs improvement to be comparable with the one achieved with cv. 60444. Certainly, the strategy of empirically finding a suitable culture media composition to produce target specific embryogenic structures of recalcitrant varieties can be a tedious and delayed process. More research is then required to optimize the efficiency of tissue culture-based methods, but at the same time, to move forward development of genotype-independent protocols for DNA-delivery and plant recovery (Altpeter et al., 2016; Baltes et al., 2017; Mookkan et al., 2017). The latest will also contribute to reducing time, costs and gaps in access to technology.

*In vitro* somatic embryogenesis (SE) is affected by many factors including genotype, explant type, growth conditions, and plant growth regulators (PGRs), among others (Zimmerman, 1993; Mordhorst et al., 1997; Jiménez, 2005). This process is highly regulated by genetic and epigenetic factors that mediate the dedifferentiation of somatic cells and the following reacquisition of totipotency (Ikeuchi et al., 2013; Feher, 2015). For cassava, attempts of addressing the genotype-dependency of FEC from a molecular perspective started to be understood through the analysis of differential gene expression between somatic embryos and FEC (Ma et al., 2015). In spite of this knowledge, the mechanisms that allow some *in vitro* cassava explants to undergo dedifferentiation, gene expression reprogramming and somatic embryo development remain unknown. Previous studies in cassava have identified proteins associated with primary and secondary SE linked to a wide range of metabolic functions (Baba et al., 2008; Li et al., 2010). However, up to date, there are no reports identifying embryo-specific regulatory genes for cassava.

In Arabidopsis the LAFL network [acronym that groups the AFL clade of B3 domain proteins and two LEC1-type HAP3 transcription factors (TFS)] is in charge of controlling seed development through the interaction of complex hormonal and intrinsic developmental signals (Jia et al., 2013). The LEAFY COTYLEDON (LEC) TFs LEC1, LEC2, and FUS3 are part of this network and have been identified as master regulators of plant embryogenesis (Braybrook and Harada, 2008). LEC TFs are responsible for establishing the cellular environment that promotes seed embryo development during both morphogenesis and maturation phases of zygotic embryogenesis (ZE) (Harada, 2001; Braybrook and Harada, 2008). Within these three genes, *LEC1* and *LEC2* play a central role in controlling many aspects of early and late embryogenesis. *LEC1* encodes a TF homolog to the HAP3 subunit of the CCAAT binding factor (CBF) (Lotan et al., 1998), while *LEC2* encodes a B3 DNA-binding motif specific to plants, which recognizes conserved RY motif and transcriptionally regulates ZE-specific genes (Stone et al., 2001; Braybrook et al., 2006). Their expression patterns are restricted to seed development and occur in a similar fashion in both ZE and SE (Lotan et al., 1998; Stone et al., 2001; Boulard et al., 2017). Loss-of-function mutations of *LEC1* and *LEC2* result in pleiotropic effects on embryo development, especially in the regulation of late embryogenesis. Mutants showed loss of embryo organs identity, intolerance to desiccation, defective storage and reserve accumulation and premature postgerminative development (Meinke, 1992; Meinke et al., 1994; Parcy et al., 1997; Harada, 2001). Moreover, when ectopically overexpressed *LEC1* and *LEC2*, separately, they were able to induce embryo development in vegetative cells, triggering the formation of embryo-like structures and the accumulation of embryo-specific RNAs (Lotan et al., 1998; Stone et al., 2001). This finding highlights the importance of LECs genes for SE.

The complex interactions between the LAFL network and hormone signaling pathways provide evidence about their key function in embryo development. Particularly, *LEC1* and *LEC2* are involved in the activation of a set of YUCCA genes (YUC family of flavin monooxygenases) that participates in the auxin biosynthetic pathway (Stone et al., 2008; Junker et al., 2012; Wójcikowska et al., 2013). Furthermore, these genes also play a role in the hormonal balance control between abscisic acid (ABA) and gibberellic acid (GA), necessary for the promotion of the maturation phase (Gutierrez et al., 2007; Yamaguchi et al., 2007). The balance between high ABA and low GA levels allows the activation of seed protein genes, responsible for storing macromolecules that are pivotal in desiccation tolerance and seedling nutrition (reviewed by Braybrook and Harada, 2008).

The key role of LEC TFs in embryogenesis regulation in Arabidopsis suggests that these genes may also be involved in the activation of SE in cassava. If true, they could provide an alternative pathway to overcome SE dependency on genotype and FEC production. It has been demonstrated that somatic cells overexpressing these genes could generate SE. Here, we characterized the Arabidopsis orthologous genes *LEC1* and *LEC2* in cassava (*MeLEC1/MeLEC2*). Changes in expression patterns were analyzed at different developmental stages during SE, and their independent overexpression validated their gene function. Finally, molecular and morpho-histological evidence of SE on leaves of *in vitro* plants supported the functional role of the analyzed *LEC* genes in embryogenesis contributing to our understanding of embryo development regulation in cassava.

## Materials and Methods

### Identification of *LEC1* and *LEC2* Orthologous Genes in Cassava

Full-length protein sequence of Arabidopsis *LEAFY COTYLEDON (LEC)* genes *LEC1* (At1g21970) and *LEC2* (At1g28300) from The Arabidopsis Information Resource (TAIR^1^) was used as a query to perform local alignments against cassava proteome using the BLASTP 2.2.26+ tool from Phytozome database^2^ (Phytozome v9.1). The obtained amino acid sequences were selected using an *E*-value cut off of 1e^-5^ and aligned using MUSCLE software in each case. Phylogenetic trees were constructed with MEGA 6.06 using the neighbor-joining method with Poisson correction, pairwise deletion and 1,000 bootstrap replicates. In the case of LEC1, 10 Arabidopsis HAP3 (HAP3) subunits were included within the phylogenetic tree together with the cassava genes candidates. The classification of LEC1-like and non-LEC1-type proteins was performed according to Kwong et al. (2003). For LEC2, proteins from Arabidopsis, containing B3 domains were included and analyzed for conserved amino acids and classified following the previous investigations of Romanel et al. (2009) and Zhang et al. (2014).

### Induction of Primary and Secondary Somatic Embryogenesis

*In vitro* cassava plants of cv. 60444 were obtained from the germplasm collection at CIAT. The composition of all media is described in Supplementary Table S1. The pH of media was adjusted to 6.12-6-13 before autoclaving (121°C at 15 PSI for 20 min). Plant material was propagated by planting cuttings of 2–3 cm long on plant propagation medium (PPM) and placed under 16/8 photoperiod at 28°C under controlled conditions. Thirty-five days old plantlets were defoliated and 3 days later axillary buds (ABs) were dissected from nodal explants and placed immediately to float on liquid-embryo induction medium (*L*-EIM). ABs were incubated in the dark at 28°C. After 21 days, primary somatic embryos were transferred onto solid embryo induction medium (*S*-EIM) under dim light at 28°C to promote secondary embryogenesis for extra 29 days. After 50 days, secondary embryos were transferred to PPM medium to induce embryo maturation. Few 50-day-old clusters were used to produce FEC according to Taylor et al. (2012).

### *MeLEC1* and *MeLEC2* Relative Expression Analysis by qRT-PCR

**FIGURE 1:**
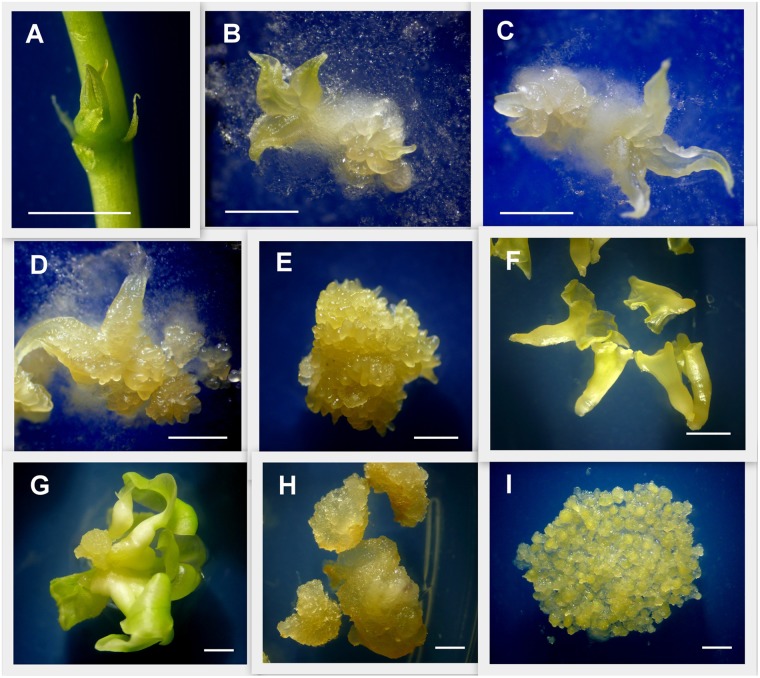
Detail of tissues used for gene expression analysis. Induction of primary and secondary somatic embryogenesis from cassava axillary buds (ABs). **(A)** ABs after 3 days of plant apex and leaves removal. **(B)** ABs after 7 days placed on embryo induction medium (*L*-EIM) forming embryogenic structures. **(C)** Fourteen days after induction (DAI). **(D)** 21 DAI. **(E)** 50 DAI, organized embryogenic structures (OES) formed by secondary somatic embryos (SEE) including globular, heart, and early-torpedo stages. **(F)** Immature cotyledonary embryos (ICE) at 50 DAI. **(G)** Mature somatic embryos 75 DAI. **(H)** Non-embryogenic callus (NEC) isolated from OES 50 DAI. **(I)** Highly pure friable embryogenic callus (FEC) produced for cassava genetic transformation. Scale bars: 2 mm.

To assess *MeLEC1* and *MeLEC2* gene expression patterns in cassava’s SE, samples of embryogenic tissues were collected at 0, 7, 14, 21, 50, and 75 days after induction (DAI) and stored in liquid nitrogen (Figure 1). Explants at 0 days were represented by dissected ABs. 50 DAI samples were dissected under the stereoscope and separately stored into secondary somatic embryo clusters (SSE, including globular, heart and torpedo stages) and immature cotyledonary embryos (ICE) derived from primary somatic embryos (Figure 1). Non-embryogenic calli (NEC) were also collected from 50 DAI clusters. Mature leaves (ML) and mature stems (MS) were sampled from 30–40 days-old *in vitro* plants and used as a source of differentiated somatic tissue. Highly pure FEC was also collected for qRT-PCR analysis. RNA was extracted using RNeasy© Plant Mini Kit (Qiagen, United States) according to manufacturer’s instructions. RNA quality and quantity was measured using a NanoDrop spectrophotometer (Thermo Scientific, United States) and was further visualized in 1.2% agarose gel. DNA contamination was avoided by treatment with DNase I Amplification Grade Kit (Invitrogen, Carlsbad, CA, United States) following manufacturer’s instructions. 1 μg of pure RNA was taken for cDNA synthesis using Transcriptor Universal cDNA Master© (Roche, Diagnostics), following manufacturer’s protocol in a 20 μl final reaction volume. All cDNAs were diluted 1:5 in DNase, RNase-free water and stored at -20°C until used. Complete *MeLEC1* and *MeLEC2* CDS sequence reported in Phytozome database were used for primer design employing the Vector NTI^®^ software version 10.3.0 (Invitrogen, Carlsbad, CA). All primer sets used in qRT-PCR analysis are indicated in Supplementary Table S2. *MeLEC1* primer set amplified a 158-bp transcript fragment and *MeLEC2* primer set amplified a 102-bp transcript fragment. Two reference genes were used to normalize qRT-PCRs measurements: *MeActin* with 170-bp transcript fragment and *Meβ-Tubulin* with 155-bp transcript fragment. Reference genes were properly tested to ensure that their gene expression was unaffected by the experimental treatment. qRT-PCR was performed in a 10 μl reaction mixture containing 1 μl diluted cDNA, 5 μl SsoAdvanced^TM^ Universal SYBR^®^ Green Supermix Kit (Bio-Rad) and 0.5 μl of each 10 μM primers following the program: 30 s at 95°C, 45 cycles of 10 s at 95°C for denaturation and 30 s at 55°C for annealing/extension. Primer pair specificity was examined previously by PCR, visualized on 1.2% agarose gel, as well as by their product qRT-PCR melting curves. Target genes were amplified from three biological replicates for each induction time (0–75 days and NTC controls) and with three technical replicates each one. Bio-Rad CFX Manager© software V3.0 (2012© Bio-Rad Laboratories) was used to calculate the C_t_ and all data was further analyzed using the 2^-ΔΔCt^ method as described by Livak and Schmittgen (2001) using the qBase software v1.3.5 (Hellemans et al., 2007). Student’s *t*-test was performed to determine significant differences (*p* < 0.01) between treatments.

### *MeLEC1* and *MeLEC2* Cloning and Expression Vector Construction

To overexpress *MeLEC1* and *MeLEC2* constitutively in cassava plants, both genes were placed independently under the control of the 2X CaMV 35S promoter. *MeLEC1* and *MeLEC2* full-length CDSs were amplified from 50 DAI cDNA samples with a specific primer set (Supplementary Table S2) and using the Phusion^®^ High-Fidelity DNA Polymerase (NEB). Both sequences were first cloned into pENTR^TM^/D-TOPO^®^ vector (Invitrogen) and sequenced, and then transferred independently into pMDC32 destination vector (Curtis and Grossniklaus, 2003) through LR reaction using the Gateway^®^ LR Clonase^TM^ enzyme mix kit (Invitrogen). Binary expression vectors were sequenced to confirm the cloning reaction and then, they were introduced into *A. tumefaciens* by heat-shock transformation.

### *Agrobacterium*-Mediated Genetic Transformation

*Agrobacterium* strain EHA105 harboring the plasmid pMDC32MeLEC1 or pMDC32MeLEC2 were used to transform cassava cv. 60444 according to Bull et al. (2009) with some modifications. Five milliliters of YEP medium with 30 μg/ml rifampicin and 50 μg/ml kanamycin were inoculated with a colony of each Agro-plasmid combination and cultured in a rotatory shaker at 220 rpm in darkness at 28°C for 6 h. 500 μl of culture was used to inoculate 20 ml YM medium with the same antibiotics and incubated overnight (16–18 h) under the same conditions. Agro-culture was grown to an OD_600_ of 0.5, centrifuged and resuspended in liquid GD2-50Pi supplemented with 200 μM acetosyringone (AS). Highly pure FEC was exposed to *Agrobacterium* for 3 days on GD2-50Pi solid medium supplemented with AS 200 μM at 21°C and 16/8 photoperiod. FEC was then washed and placed on resting medium GD2-50Pi supplemented with 250 mg⋅l^-1^ Cefotaxime. Transformed tissue was transferred to proliferation/selection media GD2-50Pi supplemented with 5 mg⋅l^-1^ Hygromycin B for 21 days at 28°C and subsequently to GD-50Pi 20 mg⋅l^-1^ hygromycin for extra 21 days. Embryos were maturated in MS2 5 μM NAA with a 16/8 photoperiod for 30 days and subcultured in MS2 0.5 μM NAA in darkness for 14 days for root and shoot elongation. Green cotyledon-stage embryos were transferred to germination medium MS2 2 μM BAP. Rooting selection was applied for final screening of regenerated plantlets using MS2 medium with 5 mg⋅l^-1^ Hygromycin B.

### Molecular Characterization of Transgenic Plants: PCR, Southern Blot, and qRT-PCR

All putative independent events were first screened by conventional PCR amplifying a fragment of the *hpt*II marker gene present in pMDC32MeLEC1 and pMDC32MeLEC2. Then, Southern blot analysis was carried out on positive-PCR plants with normal and differential phenotype. DNA extraction was conducted from leaves of *in vitro* plantlets according to Lorieux M. (pers. comm.). 10 μg of genomic DNA was digested with restriction enzyme *Kpn*I and separated by 1% agarose gel and further transferred onto a nylon membrane (Amersham Biosciences, Piscataway, NJ, United States). Membranes were hybridized at 42°C with a PCR probe synthesized with DIG DNA Labeling Kit (Roche) corresponding to a 587 bp sequence of the *hpt*II, which was amplified with the primer set *hpt*II-FW and *hpt*II-RV. Hybridization and detection were performed following manufacturer’s instructions using CDP-star (Roche Applied Science, Indianapolis, IN, United States). Once T-DNA insertion was confirmed, three independent lines for each plasmid with abnormal phenotype were analyzed by qRT-PCR. Fourth genes related to embryogenesis were included within the relative expression analysis using the same parameters employed in the previous qRT-PCR. Amplification data were normalized against *MeActin* and *Meβ-tubulin* reference genes.

### Histology

Leaf pieces from one *in vitro MeLEC2* overexpressing line were sliced and fixed in FAA solution [50% ethanol, 10% formaldehyde, 5% glacial acetic acid (v/v), 18:1:1] for 24 h. The samples were dehydrated in a graded series of 30, 50, 70, 90, 95, and 100% ethanol for 2 h, applying vacuum at each step for 5 min, finalizing with 100% butanol for 24 h. Then, each slice was embedded in butanol:resin (1:1) for 24 h and later in 100% resin [Technovit 8100 resin (Kulzer, Germany) for 48 h]. The blocks were sectioned into 3 μm thick slices using a Reichert 820H Microtome. The slices were stained with toluidine blue (0.05%) for 3 min. Images were acquired using a microscope Leica DM500.

### Scanning Electron Microscopy SEM

Leaves from one *in vitro MeLEC2* overexpressing line were observed using the microscope FEI-QUANTA 250 (United States) Universidad de Caldas-Colombia. This microscope uses the Environmental Scanning Microscope-ESEM to observe hydrated samples directly with no previous special preparation.

## Results

### Identification of *LEC1* and *LEC2* Orthologous Genes in Cassava

**FIGURE 2:**
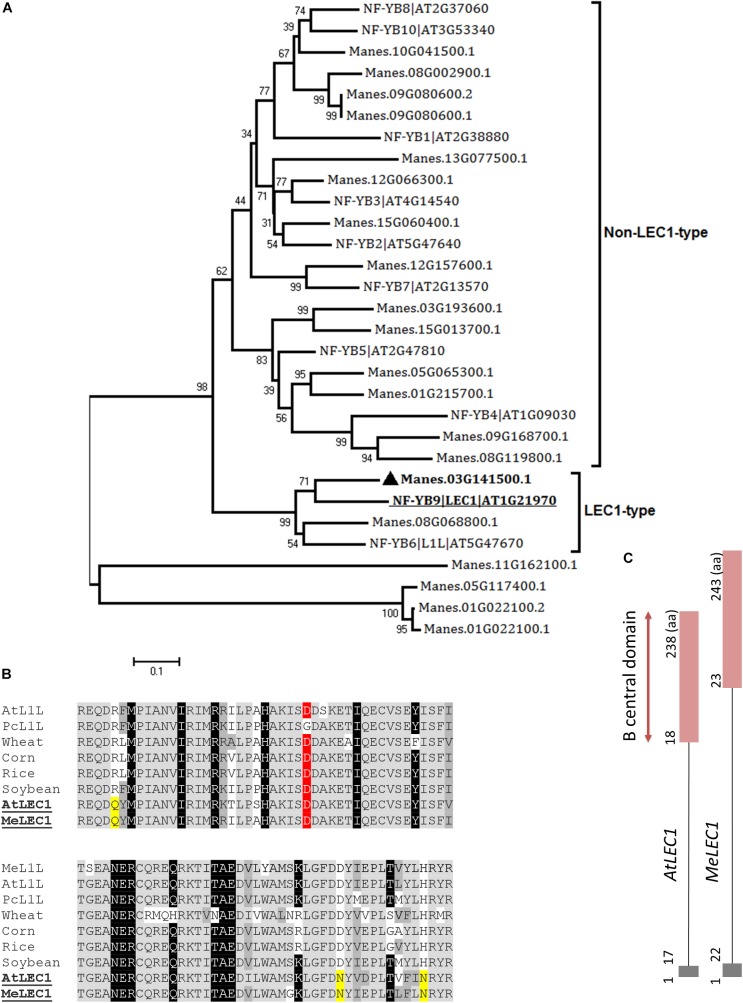
Global alignments between Arabidopsis HAP3 (AHAP3) subunits and cassava’s protein sequences and gene structure of cassava’s *LEC1* orthologous gene. **(A)** Neighbor-joining tree of the AHAP3 protein sequences and the highest score cassava’s protein sequences resulted from local alignments. Scale bar indicate 0.1 estimated substitutions per residue. Values next to the nodes show bootstrap values from 1,000 replicates. The analysis separates LEC1-type from non-LEC1-type subunits according to Kwong et al. (2003). The most closely related protein to Arabidopsis LEC1 (AtLEC1, underlined) in cassava were designed as MeLEC1 (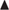). **(B)** B domain of HAP3 subunit of the CAAT binding transcription factor present in different plant species. Highlighted in gray and dark gray are indicated the conserved and similar amino acids, respectively. In black, residues shared among LEC1 and L1L-type HAP3 subunits (Kwong et al., 2003). In yellow, residues shared only between LEC1-type subunits of Arabidopsis and cassava (underlined), which differ from the L1L B domains of other plants species. Conserved Asp (D) residue within LEC1-type B domain is highlighted in red. **(C)** Comparison of *AtLEC1* and *MeLEC1* gene structures. Boxes and lines indicate exons and introns, respectively. Amino acids (aa) and B domain are also indicated. Sequences used in the alignment: *Manihot esculenta* L1L (MeL1L) Manes.08G068800.1; *Arabidopsis thaliana* LEC1-LIKE (AtL1L) AT5G47670; *Phaseolus coccineus* (PcL1L) AF533650; wheat AY058921; corn AF410176, rice AU088581, soybean AY058917, AtLEC1 At1g21970 and MeLEC1 Manes.03G141500.1.

Full-length amino acid sequences of Arabidopsis LEC1 (At1g21970) and LEC2 (At1g28300) were used as queries to identify the homologous in cassava proteome using the BLASTP algorithm and *E*-value cut-off of 1e^-5^. In the case of LEC1, blast results showed 20 possible candidate-related proteins. In Arabidopsis, LEC1 shares significant sequence similarity with the HAP3 subunit of the CCAAT binding factor (NF-Y), typically recognized by the presence of the B central domain (Lotan et al., 1998). Of the 10 subunits, two are classified as LEC1-type proteins and are involved in embryogenesis. We identified both closely related peptides in cassava. According to the neighbor-joining analysis, the protein with the best alignment to AtLEC1 corresponded to Manes.03G141500.1, hit with best score and *E*-value of 4e^-62^ (Figure 2A). This gene was designated as *MeLEC1*. Because the central B domain is the most conserved region within the HAP3 subunits, global alignments were carried out using ∼90 amino acid regions to compare MeLEC1 with other LEC1-Type subunit regions previously reported. Figure 2B details conserved and similar residues shared within the B domain from different plant species. It also highlights the amino acids shared exclusively between AtLEC1 and MeLEC1, which differ from described LEC1-Like (L1L) in accordance with Kwong et al. (2003). MeLEC1 peptide sequence consisted of 243 amino acid residues and its gene structure is detailed in Figure 2C.

**FIGURE 3:**
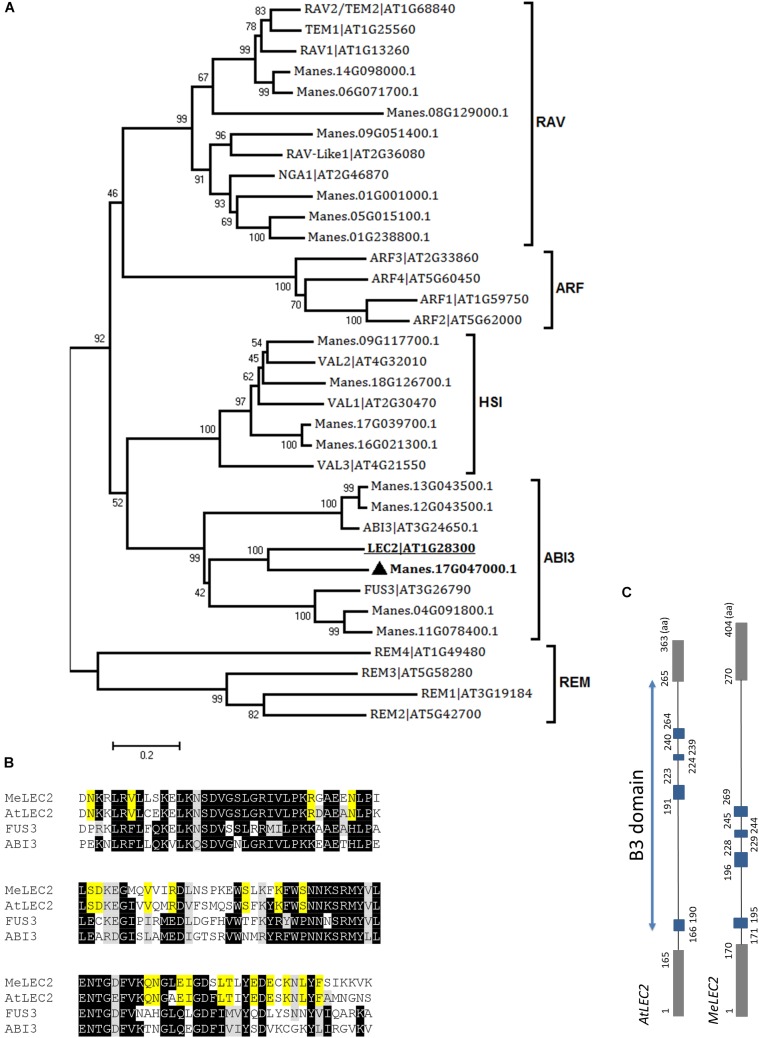
Global alignments between Arabidopsis B3 DNA binding domain (B3-BD) proteins and cassava’s protein sequences and the gene structure of cassava’s *LEC2* orthologous gene. **(A)** Neighbor-joining tree of the Arabidopsis B3-BD proteins and the highest score cassava’s protein sequences resulted from local alignments. Scale bar indicate 0.2 estimated substitutions per residue. Values next to the nodes show bootstrap values from 1,000 replicates. The analysis indicates cassava proteins related to members included in the five B3-BD families. The most closely related protein to Arabidopsis LEC2 (AtLEC2, underlined) in cassava were designed as MeLEC2 (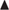). **(B)** B3 domain residues alignment from AtLEC2, AtFUS3, and AtABI3 related to MeLEC2. Highlighted in black and gray are indicated identical and similar conserved amino acids, respectively. In yellow, residues shared only between AtLEC2 and MeLEC2. **(C)** Comparison of *AtLEC2* and *MeLEC2* gene structures. Boxes and lines indicate exons and introns, respectively. B3 domain is indicated in blue rectangles. Amino acids (aa) are also indicated. Sequences used in the alignment: AtFUS3 At3g26790; AtABI3 At3g24650; AtLEC2 At1g28300, and MeLEC2 Manes.17G047000.1.

In the case of LEC2, 16 related proteins were found in cassava. In Arabidopsis, *LEC2* encodes a B3 DNA binding domain (B3-BD) TF. This B3-BD is found in five gene families such as auxin response factor (ARF), abscisic acid-insensitive 3 (ABI3), high-level expression of sugar inducible (HIS), related to ABI3/VP1 (RAV), and reproductive meristem (REM). Members of the five families were used to perform global alignments with resulted cassava candidate genes. We were able to identify closely related peptides within the classified families. According to the neighbor-joining analysis, the protein with the best alignment to AtLEC2 corresponded to Manes.17G047000.1, hit with the best score and *E*-value of 2.5e^-57^ (Figure 3A). This gene was designated as *MeLEC2*. Global alignments were carried out between MeLEC2 B3-BD and the most similar TFs of AtLEC2 containing a B3-BD, which included AtFUS3 and AtABI3. MeLEC2 B3-BD shared 75, 50, and 51% amino acid identity with AtLEC2, FUS3, and ABI3, respectively. Residues shared only between AtLEC2 and MeLEC2 were also discriminated (Figure 3B). MeLEC2 peptide sequence consisted of 405 amino acid residues and its gene structure is detailed in Figure 3C.

All these *in silico* analyses presented above provides evidence that suggests that unique homologous sequences of AtLEC1 and AtLEC2 are present in cassava. MeLEC1 and MeLEC2 sequences were then used to perform the subsequent expression analyses.

### *MeLEC1* and *MeLEC2* Are Highly Expressed During the Induction of SE

To detect *MeLEC1* and *MeLEC2* mRNA levels we induced embryogenic callus and subsequently somatic embryos. SE induction was performed by using ABs from adult plantlets placed onto Picloram-auxin supplemented medium. Seven DAI, the growth of first pro-embryo structures were observed together with a thin layer of cells surrounding the explants (Figure 1B). 50 DAI explants developed into organized embryogenic structures (OES) formed by secondary somatic embryos (SSE), which included stages from globular to torpedo. OES also harbored ICE, which clearly differed from SSE in their green cotyledon-like structure, and because of this, we decided to sampled them independently.

**FIGURE 4:**
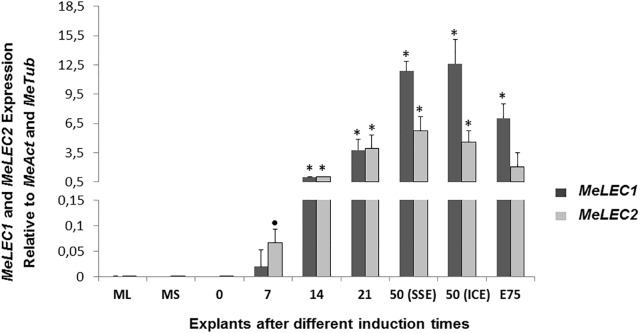
Relative expression patterns of *MeLEC1* and *MeLEC2* genes during SE induction and the subsequent development of cassava somatic embryos. Measurements were normalized against *MeActin* and *Meβ-tubulin* reference genes. Samples at 0, 7, 14, 21, 50 (SSE, secondary somatic embryos), 50 (ICE, immature cotyledonary embryos) and 75 days after induction were analyzed. Mature leaves (ML) and mature stems (MS) were also included in the analysis. 14 DAI were chosen as calibrator. Each reaction was conducted three times with three independent biological replicates (*n* = 3). Error bars correspond to the normalized ± SD of 2^-ΔΔCt^ values. Significance was established by *t*-test. ^∗^ Represents significant difference between the compared induction times (0–75) and the 0 DAI sample, *p*-value < 0.01. (•) Represents *p*-value < 0.05 by *t*-test.

**FIGURE 5:**
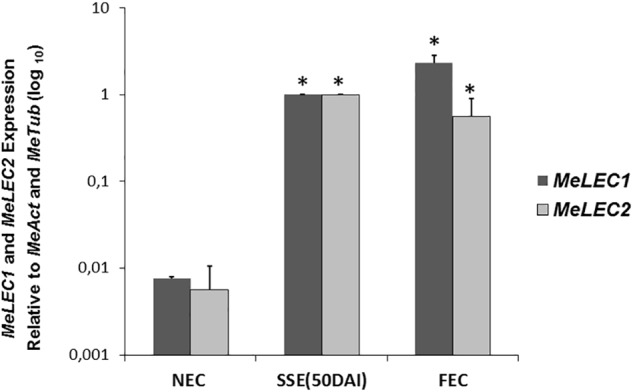
Relative expression profiles of three types of *in vitro* induced callus. NEC, non-embryogenic callus. SSE (50 DAI), secondary somatic embryos 50 days after induction. FEC, friable embryogenic callus. Measurements were normalized against *MeActin* and *Meβ-tubulin*. Each reaction was conducted three times with three independent biological replicates (*n* = 3). SSE (50 DAI) was chosen as calibrator. Error bars correspond to the normalized ± SD of 2^-ΔΔCt^ values. *p-*value < 0.05 was established by *t*-test. ^∗^ Represents significant difference compared to NEC.

Tissue samples were collected during SE induction at different times (Figure 1) and three biological replicates were analyzed in qRT-PCR experiments. Relative expression analyzes showed that *MeLEC1* and *MeLEC2* transcripts increased exponentially with the advance of SE (Figure 4). Significant differences were found when the expression levels at each time were compared with those detected at 0 DAI. *MeLEC1* and *MeLEC2* transcript changes were detected as early as 7 DAI. Until 21 days *MeLEC2* expression levels appeared to be slightly higher than *MeLEC1*, but as with SE progress, *MeLEC1* levels increased differentially with respect to *MeLEC2* (Figure 4). We also compare the gene expression levels found in FEC and secondary somatic embryos (SSE) at 50 DAI vs. non-embryogenic callus (NEC) generated at the same age. The expression profile of SSE (50 DAI) denoted the presence of both *MeLEC1* and *MeLEC2* with approximately 100-fold increment when they were contrasted with NEC (Figure 5).

Collectively these findings suggest that *MeLEC1* and *MeLEC2* are performing a role in SE since the beginning of the process until when maturation of the somatic embryos is in course. Apparently, they are not required for vegetative growth. Additionally, even though the production of different callus types is a consequence of the exposure of differentiated tissue to auxin-containing media, the activity of both genes seems to be related only with embryogenic callus and with cells/tissue with totipotent characteristics.

### Ectopic Overexpression of *MeLEC1* and *MeLEC2* Contributed to Elucidate Their Role in SE

To demonstrate whether the overexpression of *MeLEC1* and/or *MeLEC2* in somatic tissues of cassava was enough to induce SE from vegetative cells, their coding sequences were cloned independently into a binary expression vector driven by the 35S promoter. Each construct was used to transform FEC to produce transgenic cassava lines overexpressing both genes independently. Somatic embryos were recovered after transformation. In the case of *35S:MeLEC1*, there were normal and abnormal embryos that frequently occur because of somaclonal variation. In case of *35S:MeLEC2* somatic embryos, striking morphologies were registered in some of them like the absence of apical meristems; overgrow of cotyledon-like structures and, in some cases, an absence of root development (Supplementary Figure S1). Additionally, the presence of embryo-like structures emerging over their cotyledon surface was noted. Some of these abnormal embryos continued growing on media but were not be able to convert to plants. Because no differences were observed in cassava’s *35S:MeLEC1* at embryo stages that provided evidence about the effect of *MeLEC1* overexpression, Arabidopsis was transformed with the same *35S:MeLEC1* construct using the floral dip method (Zhang et al., 2006). Plantlets with arrested development and embryo morphology were registered. Moreover, the first true leaves with cotyledon-like appearance and the occurrence of callus-like structures emerged on selection media without PGRs were also observed (Supplementary Figure S2). The presence of the transgene was detected and semi-quantified by RT-PCR.

**FIGURE 6:**
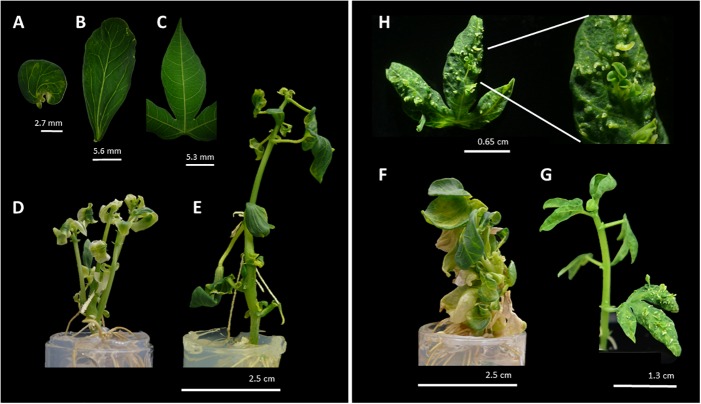
Phenotype of regenerated *MeLEC* overexpressing lines (*MeLEC^OE^*). *MeLEC1^OE^* round **(A)** and elongated **(B)** cotyledon-like matures leaves with abnormal vascular vessel pattern. **(C)** Normal leaf of cassava *in vitro* plant. Examples of plant architectures obtained after *MeLEC1^OE^*: **(D)** Regenerated plant with multiple shoots and abnormal leaves with reduced petioles. **(E)** Regenerated plant harboring leaves indicated in **(A,B)**. Examples of plant architecture obtained after *MeLEC2^OE^*. **(F)** Regenerated plant with round and fleshy leaves, and reduced apical meristem and petioles. **(G)** Regenerated plant with somatic embryo-like structures over the entire surface of differentiated leaves **(H)**.

Following regeneration process, a total of 140 *35S:MeLEC1* and 125 *35S:MeLEC2* plants were recovered. Leaf pieces of 104/140 (74%) of *35S:MeLEC1* and 66/125 (53%) of *35S:MeLEC2* plants were used for DNA extraction and PCR analysis. As expected 100% of the plants were PCR-positive (data not shown). Since the beginning of regeneration, some of the transgenic plantlets showed remarkable morphological changes, but most of them recovered their normal appearance after several cycles of *in vitro* propagation. After three cycles of propagation, 35/140 (25%) of *MeLEC1* and 13/125 (10%) of *MeLEC2* plants retained the abnormal phenotype. In the case of *MeLEC1* overexpressing (*MeLEC1^OE^*) lines, it was observed the presence of mature leaves that acquired the cotyledon-shape with a different vascular vessel pattern (Figure 6A–E). In some cases, multiple shoots were observed, with cotyledon-like leaves, almost with no petioles (Figure 6D). In the case of *MeLEC2^OE^* the remaining abnormal lines showed two contrasting phenotypes, one type (Figure 6F) with round-fleshy leaves, and no apparent internodes and petioles; and a second type (Figure 6G), close to normal vegetative growth but with embryo-like structures emerging over the entire leaf surface (Figure 6F,H). This feature was registered in at least three *MeLEC2^OE^* lines, and it was consistently present in line L2.1. Some normal and abnormal phenotype overexpressing lines for each gene were analyzed by Southern blot. Results indicated no correlation between T-DNA insertions and the observed phenotypes (Supplementary Figure S3).

### *MeLECs* Overexpression Triggers SE Transcription Programs in Cassava Vegetative Tissues

**FIGURE 7:**
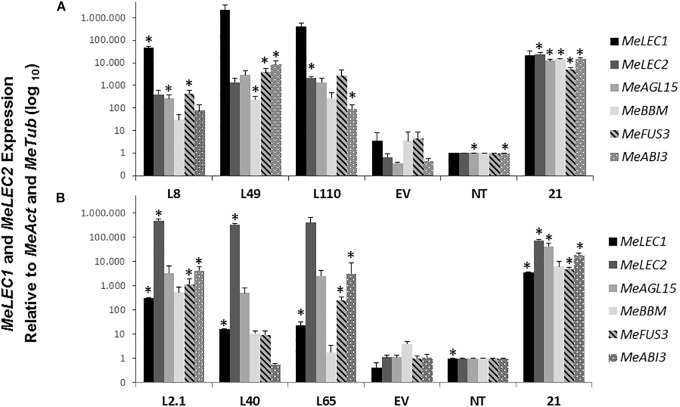
Relative expression analysis of somatic embryogenesis-related genes induced in overexpressing MeLEC transgenic lines. Expression levels of *MeLEC1, MeLEC2, MeAGL15, MeBBM, MeFUS3*, and *MeABI3* were measured within three *35S:MeLEC1* transgenic lines: L8, L49, and L110 **(A)** and three *35S:MeLEC2* transgenic lines: L2.1, L40, L65 **(B)** and were normalized against *MeActin* and *Meβ-tubulin*. Each reaction was conducted three times with three independent biological replicates (*n* = 3). Non-transgenic (NT) control was chosen as calibrator. Error bars correspond to the normalized ± SD of 2^-ΔΔCt^ values. *p-*value < 0.05 was established by *t*-test. ^∗^ Represents significant difference compared to empty vector control (EV). 21, embryogenic clusters 21 days after induction.

To investigate whether the morphological effect produced by the overexpressing *MeLEC* genes was related to SE, we measured mRNA levels of the following, well-characterized embryo-regulatory factors in Arabidopsis (Supplementary Table S2): AGAMOUS-Like 15 (AGL15) which is a MADS-box TF directly regulated by LEC2 that promotes competence for SE by repressing the levels of GA (Wang et al., 2004). BABY BOOM (BBM) which is also a TF that regulates the expression of key factors for embryo maturation and SE initiation including *LEC1, LEC2, ABI3*, and *FUS3* (Horstman et al., 2017). Finally, FUSCA 3 (FUS3) and ABSCISIC ACID INSENSITIVE 3 (ABI3) were measured at mRNA level, both are B3-BD TFs which interact with LEC TFs to regulate the seed maturation phase of embryo development (Luerßen et al., 1998; Mönke et al., 2004). Protein sequences of those genes were used to find the homologous genes within cassava genome, and similar approaches as for MeLEC1 and 2 were conducted to choose the most closely related sequences. Three independent *MeLEC^OE^* lines with abnormal phenotypes were analyzed by qRT-PCR. Results indicated that in fact, MeLEC transgenic plants were overexpressing either *MeLEC1* or *MeLEC2* genes when compared with the empty vector or with the non-transgenic controls (Figure 7). At the same time, a high induction of all other embryo-associated genes was detected and in some of the cases, significant differences were established. For *MeLEC1^OE^* lines, the embryo-associated genes were overexpressed in similar fashion among them. In *MeLEC2^OE^* lines, the expression patterns resulted less similar, with particular attention on line L2.1, in which the expression levels of the embryo-associated genes were relatively higher. However, the patterns observed in both MeLEC1 and MeLEC2 overexpressing lines, they were quite different from those found in embryogenic clusters samples, where the six genes were highly active.

Overall, these results showed that the independent overexpression of *MeLEC1* and *MeLEC2* genes was able to upregulate the expression of other embryo-specific TF, with which normally they are coexpressed, providing evidence about their mutual regulation that enables the embryo development.

### *MeLEC2* Overexpression Induces Somatic Embryo Formation in Vegetative Tissue

**FIGURE 8:**
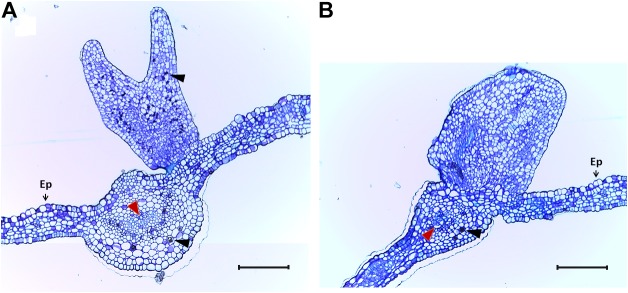
Histological sections of *in vitro* cassava leaves of *MeLEC2* overexpressing Line 2.1. **(A)** Transversal section showing a normal torpedo-stage embryo developed over the adaxial surface of mature leaf, close to the leaf mid-vein. **(B)** Transversal section showing an abnormal embryo-like mass developed over the adaxial surface of mature leaf. Red arrowheads indicate leaf vascular tissue. Black arrowheads indicate starch granules. Ep, adaxial epidermis. Scale bars: 200 μm.

Due to the remarkable characteristic of somatic embryo-like structures emerging constantly from the entire surface of leaves of the *MeLEC2^OE^* line L2.1, leaf sections were analyzed in detail by histology and SEM. Transversal sections highlighted similarities in morphology between these embryo-like structures and zygotic embryos, including cotyledons and embryo axis development, as recorded in Figure 8A. In addition, the formation of compact cell masses was detected probably arising as a result of uncontrolled cell division (Figure 8B). SEM photographs registered the formation of embryo-like structures at different developmental stages, from globular to cotyledonary embryos.

## Discussion

Cassava FEC-based GT has led the way to answer fundamental questions about crop biology, breeding and alternative propagation systems. However, the variation in embryogenic response among genotypes precludes the scaling-up of the technology and their application for more varieties. A deep understanding of SE induction regulation in cassava could contribute to improving tissue culture methods for GT and new breeding techniques (NBTs) such as GE. Results presented here describe the identification and characterization of Arabidopsis *LEC1* and *LEC2* orthologous genes in cassava, two TFs that play an important role in the regulation of embryo development. Expression analysis and overexpressing experiments revealed robust evidence that supports the participation of both TFs in the promotion of SE of cassava and their central role in the transcriptional regulation of this process.

Bioinformatics analyses supported the election of unique candidate homologs for each gene. The sequence similarity of the LEC1-type B domain shared between *MeLEC1* and other plant species, and the presence of critical amino acids suggested a highly conserved molecular function. Some typical residues of plant LEC1-LIKE (L1L) proteins allowed to differentiate from AtLEC1 and MeLEC1 proteins, and consequently identify the homolog sequence of *AtL1L* in cassava (*MeL1L*; Figure 2) as well. *AtL1L* also encodes a TF that belongs to the LAFL network, and its function is partially overlapped with *LEC1* (Kwong et al., 2003). In the case of *MeLEC2*, a single copy of the gene was found in the cassava genome, sharing high homology with Arabidopsis B3-BD proteins. To date, more *AtLEC2* homologous proteins have been identified in other plant species like *RcLEC2* (399 aa) from castor bean (*Ricinus communis*) (Kim et al., 2014) and *TcLEC2* (455 aa) from cacao (*Theobroma cacao*) (Zhang et al., 2014). They all exist as single copy genes, and their gene structure is closely related to each other.

The sustained change in gene expression levels of *MeLEC1* and *MeLEC2* during all SE induction process suggest that both TFs are actively participating in embryo development, from early morphogenesis until late maturation stages. This finding is consistent with Arabidopsis expression pattern, where the LEC TFs are exclusively expressed during embryo development. The expression of *MeLEC2* was slightly higher than the one found in *MeLEC1* in the first 21 days of SE induction, which mirrors the expression pattern of *AtLEC2* at early stages of embryogenesis (Stone et al., 2001; To et al., 2006; Boulard et al., 2017). This also suggests that *MeLEC2* is probably required in the transition from somatic to embryogenic fate. Contrary to this, in cacao, the *TcLEC2* expression levels only were detected 41 days after exposing somatic tissue to exogenous auxin, just when the first somatic embryos started to appear (Zhang et al., 2014). That is an example that demonstrates the high variation that exists within cells and plant species in acquiring the competence required to start SE. For *MeLEC1*, the transcripts were also detected since the beginning of SE induction and with high levels even at later stages. This expression pattern coincides with the one described for Arabidopsis, where *AtLEC1* is also involved in the hypocotyl elongation by mediating the auxin accumulation in the root-hypocotyl junction (Junker et al., 2012).

Constitutively overexpressing *MeLEC1* and *MeLEC2* in cassava provides fundamental insights about their function. First, in *MeLEC1^OE^* plants the normal three-lobed palmate-shaped leaves were replaced by round cotyledon-like leaves in which changes in the shape of the vascular vessels could also be registered (Figure 6A,B). This phenotype can be explained by one of the main functions of *LEC1*, which is specifying the cotyledon identity in Arabidopsis (Lotan et al., 1998). Moreover, the heterologous expression of *MeLEC1* in Arabidopsis displayed many similarities in relation to the morphological structures that were obtained when *AtLEC1* is overexpressed in the host plant, including fleshy cotyledons, swollen hypocotyl, abnormally shaped leaves and greened roots. Callus-like structures were also observed in germinated Arabidopsis embryos, showing the capacity of *MeLEC1* to induce cell division in auxin-free culture medium (Lotan et al., 1998; Junker et al., 2012). The overexpression of *MeLEC2* led to altered morphology and regeneration difficulties of somatic embryos. In Arabidopsis, the ectopic expression of *LEC2* can up-regulate *FUS3*, responsible of controlling the ABA/GA ratio in developing seeds, which is in turn required to coordinate the balance between dormancy and germination (Gazzarrini et al., 2004; Braybrook and Harada, 2008). This could explain the retention of an embryonic stage and the hurdles to overcome it to promote vegetative growth. Concomitantly, the increment on transcript levels of *MeLEC1* and *MeLEC2* were able to induce the expression of different embryo-associated TFs such as *MeAGL15, MeBBM, MeFUS3*, and *MeABI3* in vegetative tissue, demonstrating that MeLEC targets are also conserved in cassava, suggesting a mutual regulation as it occurs in *Arabidopsis thaliana* (reviewed by Jia et al., 2014). Taken together these results support the conserved phylogenetic function of *MeLEC1* and *MeLEC2* in cassava.

**FIGURE 9:**
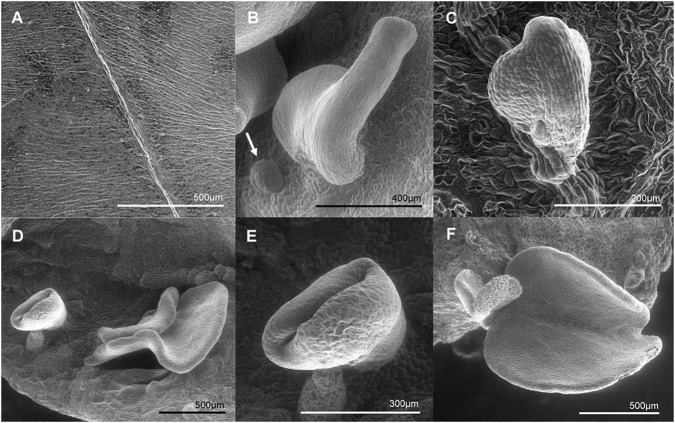
Scanning electron microscopy (SEM) photographs of somatic embryo-like structures developed over the adaxial surface of *MeLEC2^OE^ in vitro* cassava leaves of Line 2.1. **(A)** Fully expanded mature Leaf of *wt* 60444. **(B)** Somatic embryo-like emerging over the adaxial surface. Globular stage embryo is indicated by white arrow. **(C)** Heart embryo stage emerging from the mid vein of mature leaf. **(D)** Embryo-like structures at different stages developing at non-specific site. **(E)** Torpedo stage. **(F)** Cotyledon stage at the leaf margin.

The ability to produce somatic-like embryos over the surface of mature leaves in *MeLEC2^OE^* transgenic lines reveals the key function of *MeLEC2* in the reprogramming of differentiated vegetative cells. Different embryo stages, ranging from globular to early-cotyledonary shapes, were observed sharing many morphological similarities with stages that occur in ZE (Figure 8, 9). Several studies describe the generation of secondary somatic embryos mainly on the cotyledon surface and roots of Arabidopsis seedlings when morphogenic genes are ectopically overexpressed, such as *AtLEC1* (Lotan et al., 1998), *AtLEC2* (Stone et al., 2001), *Wuschel* (*WUS*; Zuo et al., 2002), *Baby Boom* (*AtBBM*; Boutilier et al., 2002), *GmBBM* (El Ouakfaoui et al., 2010) and *RKD4* (Waki et al., 2011). Particularly, the present study evidenced secondary embryos not only on somatic embryos but also on mature plants. Nevertheless, the fact that these structures were present in certain *MeLEC2^OE^* lines, and within them, only in leaves, is in agreement with the hypothesis that only certain class of cells are able to switch from vegetative to an embryonic stage (Feher, 2015), regardless of the ectopic overexpression of embryogenesis genes like *MeLEC2*. Our findings demonstrate that some of these somatic embryos emerged from different parts on the leaves, suggesting that this ability would not be restricted to a specific cell type. This result contrasts with what has been reported for secondary SE induction in cassava, where embryos arise mainly around vascular procambium (Baba et al., 2008), a region of high competence for SE. However, it is important to highlight the expression profile of line L2.1, one of the *MeLEC2^OE^* lines in which somatic-like embryos were observed. In this, the transcript levels of *MeAGL15, MeBBM, MeFUS3, MeABI3*, and *MeLEC1* were higher compared to the other two lines that did not have these structures and could resemble the pattern found in embryogenic clusters (Figure 7). This finding suggests that this type of expression profile is probably required for cell reprogramming and embryo competence acquisition and, at the same time that other factors not measured in this study are being activated by MeLEC2, responsible for this phenotype.

Like in most important crops, GT in cassava is still confined to few genotypes, usually model plants or varieties with low-yielding performance, in spite of the recent advances on this technology. New strategies are implementing the use of morphogenic regulators (MRs) or embryogenesis promoting factors (EPFs) to increase plant regeneration of recalcitrant species and leaving behind the classical approach of deciphering the suitable media composition. In monocot species such as maize, rice, sorghum and sugarcane, the use of MRs such as *WUS* and *BBM* has made possible to promote SE and to enhance the regeneration of recalcitrant genotypes during GT (Lowe et al., 2016; Mookkan et al., 2017). In this research, we demonstrated that at least *MeLEC2* is a TF that regulates and promotes SE in cassava. Modulating *MeLEC2* expression could stimulate embryo development and enhance subsequent plant regeneration of more cassava genotypes through SE. It may also reduce tissue culturing time and therefore the frequency of somaclonal variation in modified plants. Finally, manipulating the expression of these EPF may accelerate the use of GE, a technology that relies heavily on tissue culture, but that is a powerful tool to accelerate the breeding of cassava.

## Author Contributions

AB and PC-A conceived the idea. AB designed the experiments, performed the bioinformatics and expression analysis, produced and characterized the transgenic plants, and wrote the manuscript. MQ led the bioinformatics analyses and gene expression experiments. PC-A and JT supervised the design of all experiments. MQ and PC-A edited the manuscript.

## Conflict of Interest Statement

The authors declare that the research was conducted in the absence of any commercial or financial relationships that could be construed as a potential conflict of interest.

## Footnotes

1 http://arabidopsis.org

2 http://phytozome.net

## Supplementary Material

FIGURE S1 Embryo-like structures and abnormal features present on regenerated *35S:MeLEC2* cassava embryos. In some of them root development is observed, but apical meristem is absent in the majority. **(A)** Embryo after 40 days on germination medium MS2 2 μM BAP. In detail, embryo-like structures emerging from the cotyledon’s surface. **(B,C)** Embryos after 64 days on germination medium. Growth of lettuce-like cotyledons is observed, as well as secondary cotyledonary structures. **(D)** Normal empty vector transgenic embryo after 30 days on germination medium. Scale bars: 7.5 mm.

FIGURE S2 *MeLEC1* heterologous overexpression in Arabidopsis. Embryonic characteristics retained by Arabidopsis ecotype Col-0 seedlings transformed with the *35S:MeLEC1* construct using the floral dip method (Zhang et al., 2006). **(A)** Wild-type (*wt*) seedling 7 days after sowing (DAS) in MS medium containing 15 mg⋅l^-1^ hygromycin B. **(B)** *35S:MeLEC1* seedling seven DAS. Arrested vegetative development and long root grow are observed. **(C)** *wt* plant 30 DAS in selection medium. **(D)** *35S:MeLEC1* seedling 30 DAS in selection medium. Development of the first leaves similar to cotyledons. They remain fleshy and failed to expand. **(E)** *35S:MeLEC1* seedling 45 DAS. Punctual features shared with *LEC1* overexpressing seedlings (Lotan et al., 1998). FC, non-expanded and fleshy cotyledons; Cll, callus-like structures; GR, greened roots. **(F)** RT-PCR of three *35SMeLEC1* seedlings expressing *MeLEC1* and *hpt*II resistance gene.

FIGURE S3 Southern blot analysis of transgenic *35S:MeLEC1* and *35S:MeLEC2* plants. Both membranes were hybridized with the *hpt*II probe. Some putative independent transgenic lines were randomly chosen for Southern blot analysis and grouped in abnormal and normal phenotype. **(A)** For *35S:MeLEC1*, 14 and 11 lines were included as abnormal and normal, respectively. **(B)** In case of *35S:MeLEC2*, seven and eight events were included as abnormal and normal, respectively. First lane corresponds to non-transgenic *wt* 60444 and last lane to pMDC32 plasmid.

TABLE S1 Culture media used in the present study.

TABLE S2 Primer list used in the present study.
