# Supplementary figures and images for: Arabidopsis LEC1 and LEC2 Orthologous Genes Are Key Regulators of Somatic Embryogenesis in Cassava
Source: Front Plant Sci. 2019 May 22;10:673. doi: 10.3389/fpls.2019.00673 (PMC6541005; doi:10.3389/fpls.2019.00673)

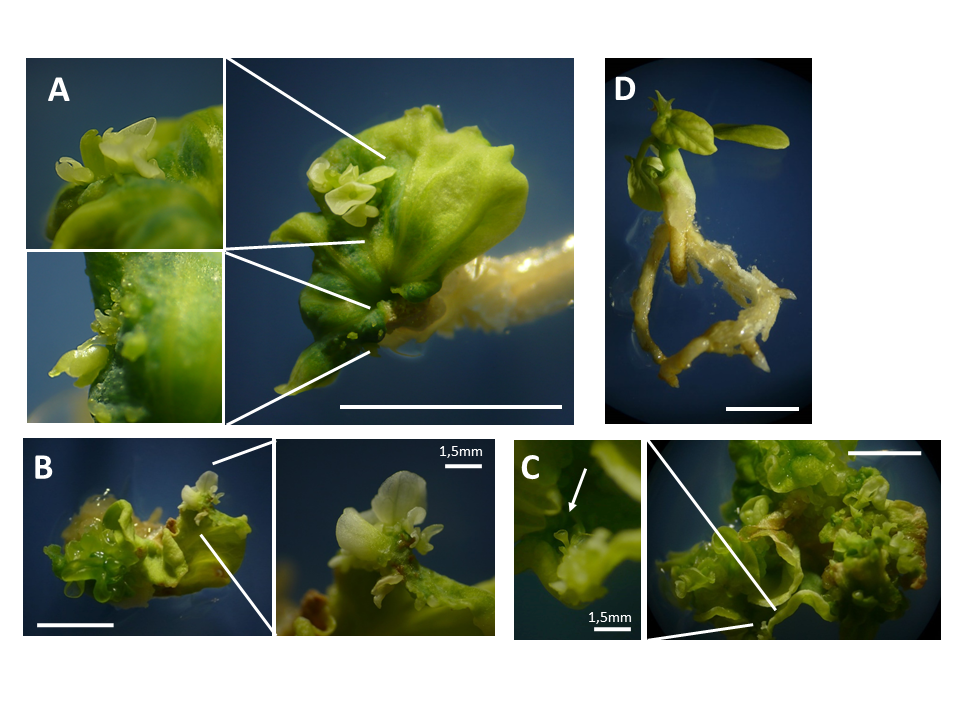

Supplement: FIGURE S1 — Embryo-like structures and abnormal features present on regenerated 35S:MeLEC2 cassava embryos. In some of them root development is observed, but apical meristem is absent in the majority. (A) Embryo after 40 days on germination medium MS2 2 μM BAP. In detail, embryo-like structures emerging from the cotyledon’s surface. (B,C) Embryos after 64 days on germination medium. Growth of lettuce-like cotyledons is observed, as well as secondary cotyledonary structures. (D) Normal empty vector transgenic embryo after 30 days on germination medium. Scale bars: 7.5 mm. [file Image_1.TIF]

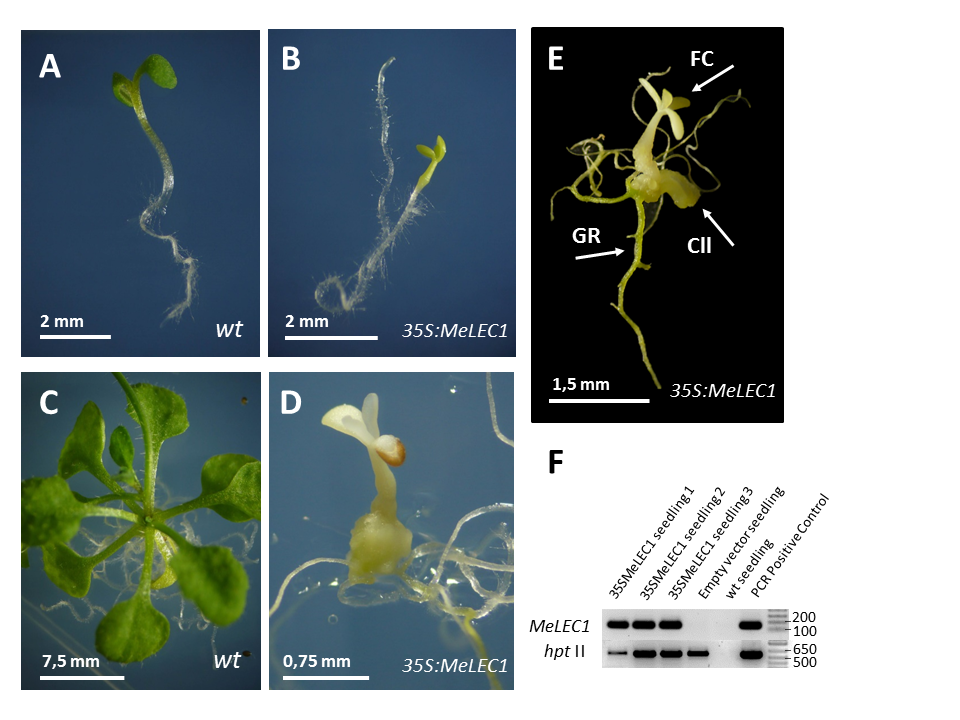

Supplement: FIGURE S2 — MeLEC1 heterologous overexpression in Arabidopsis. Embryonic characteristics retained by Arabidopsis ecotype Col-0 seedlings transformed with the 35S:MeLEC1 construct using the floral dip method (Zhang et al., 2006). (A) Wild-type (wt) seedling 7 days after sowing (DAS) in MS medium containing 15 mg⋅l-1 hygromycin B. (B) 35S:MeLEC1 seedling seven DAS. Arrested vegetative development and long root grow are observed. (C) wt plant 30 DAS in selection medium. (D) 35S:MeLEC1 seedling 30 DAS in selection medium. Development of the first leaves similar to cotyledons. They remain fleshy and failed to expand. (E) 35S:MeLEC1 seedling 45 DAS. Punctual features shared with LEC1 overexpressing seedlings (Lotan et al., 1998). FC, non-expanded and fleshy cotyledons; Cll, callus-like structures; GR, greened roots. (F) RT-PCR of three 35SMeLEC1 seedlings expressing MeLEC1 and hptII resistance gene. [file Image_2.TIF]

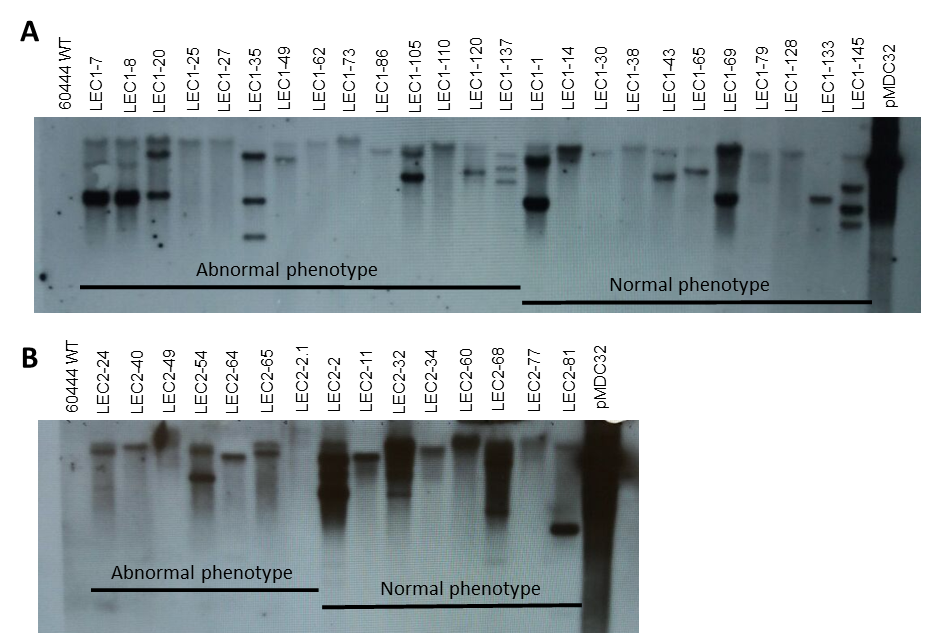

Supplement: FIGURE S3 — Southern blot analysis of transgenic 35S:MeLEC1 and 35S:MeLEC2 plants. Both membranes were hybridized with the hptII probe. Some putative independent transgenic lines were randomly chosen for Southern blot analysis and grouped in abnormal and normal phenotype. (A) For 35S:MeLEC1, 14 and 11 lines were included as abnormal and normal, respectively. (B) In case of 35S:MeLEC2, seven and eight events were included as abnormal and normal, respectively. First lane corresponds to non-transgenic wt 60444 and last lane to pMDC32 plasmid. [file Image_3.TIF]
