# Supplementary material for: Arabidopsis LEC1 and LEC2 Orthologous Genes Are Key Regulators of Somatic Embryogenesis in Cassava
Source: Front Plant Sci. 2019 May 22;10:673. doi: 10.3389/fpls.2019.00673 (PMC6541005; doi:10.3389/fpls.2019.00673)
Supplement: TABLE S1 — Culture media used in the present study. [file Table_1.docx]

**Supplementary Table 1**.

**Culture media used in the present study.**

| **Abbreviation** | **Media composition** |
| --- | --- |
| PPM | MS (Murashige and Skoog-Duchefa®) basal medium supplemented with 2% sucrose; 0,04mg·l^-1^ BAP; 0,05 mg·l^-1^ GA3, 0,02 mg·l^-1^ NAA and 2 μM CuSO4.5H2O. |
| *L*-EIM | MS (Murashige and Skoog-Duchefa®) basal medium supplemented with 2% sucrose, Gamborg’s B5 vitamins, 2 μM CuSO4.5H2O,  150 mg·l^-1^ casein hydrolysate, and supplemented with 50 μM of Picloram. |
| *S*-EIM | MS (Murashige and Skoog-Duchefa®) basal medium supplemented with 2% sucrose, Gamborg’s B5 vitamins, 2 μM CuSO4.5H2O,  150 mg·l^-1^ casein hydrolysate, supplemented with 50 μM of Picloram and 2 g·l^-1^ Gelrite. |
| GD2-50Pi | Gresshoff & Doy Micros and Macro elements and Vitamins, supplemented with 2% sucrose; FeNa-EDTA and 50 μM of Picloram. |
