# Supplementary material for: Arabidopsis LEC1 and LEC2 Orthologous Genes Are Key Regulators of Somatic Embryogenesis in Cassava
Source: Front Plant Sci. 2019 May 22;10:673. doi: 10.3389/fpls.2019.00673 (PMC6541005; doi:10.3389/fpls.2019.00673)
Supplement: TABLE S2 — Primer list used in the present study. [file Table_2.DOCX]

**Supplementary Table 2**.

Primer list used in the present study.

| **Gene** | **Locus** | **Cassava Transcript ID** | **Primer Fw** | **Priver Rv** | **Amplicon length (bp)** |
| --- | --- | --- | --- | --- | --- |
| *MeActin* |  | Manes.01G012800.1 | TCAATCCGTCCTTGGCTCAG | CATTGATGCTGTGCCACCAG | 170 |
| *MeTubulin* |  | Manes.08G061700.1 | GGAGCCTGGTACAATGGACAG | CAACATCAAGAACCGCATCG | 155 |
| *MeLEC1* | AT1G21970 | Manes.03G141500.1 | GCGTCGTATCCTCCCAGCTC | CCCAAAGAACGTCCTCAGCAG | 158 |
| *MeLEC2* | AT1G28300 | Manes.17G047000.1 | GGGCTGGAGATTGGAGATTC | GTATGACACCTCGATTTGCGG | 102 |
| *MeAGL15* | AT5G13790 | Manes.07G011600.1 | GATCACCCAGTCCCAACTTATC | GGTTGTATCCGAGTCTCCTTTC | 129 |
| *MeBBM* | AT5G17430 | Manes.13G001800.1 | CAGGAGTATGTTGCCTCTCTTC | CTTCCATGCTGATGGTGTCT | 95 |
| *MeFUS3* | AT3G26790 | Manes.11G078400.1 | TCCTCTTCGCATCACCTTTC | CTCTTCAGGGAGCTTACATCAC | 110 |
| *MeABI3* | AT3G24650 | Manes.13G043500.1 | CGGAACTGTTATTCCGGGTTAT | GATGGTGGTATTGGTGGGTAAG | 111 |
| *hpt*II-FW/RV | |  | CGTCTGCTGCTCCATACAAG | ATAGCTGCGCCGATG GTT | 587 |
| MeLEC1-10FW/MeLEC1-10RV | | | CACCATGGAACGTGGAGGCAGGCT | TTACTTGAACTGAGCAAACGGATC | 736 |
| MeLEC2-5'UTR-FW3/MeLEC2-11RV | | | CACCGATAGCGAATCAAAAATG | CTAAAAATTATACCTATTGACATC | 1234 |
